# Stewardship configuration and medicines governance under fiscal stress: lessons from Botswana's 2025 health emergency

**DOI:** 10.3389/frhs.2026.1851498

**Published:** 2026-06-19

**Authors:** Thabo Lucas Seleke, Keitseope Nthomang

**Affiliations:** 1 Department of Politics and Public Administration, University of Botswana, Gaborone, Botswana; 2 Department of Social Work, University of Botswana, Gaborone, Botswana

**Keywords:** accountability, Botswana, health systems governance, medicines supply chain, professional dominance, stewardship

## Abstract

**Background:**

Botswana's health system has been characterized in terms of its handling of HIV/AIDS and the comparability of public-sector delivery. These characterizations have pretty good bedrock. The 2025 declaration of an official public health emergency, after disruptions of the supply of almost all essential medicines, threw a spanner in the works. Public discourse changed in response. The conversation now became primarily about budgetary and supply chain procurement challenges. While these were concerns, there were several other issues, the most troubling being the absence of a systemic response to the multiple challenges and warnings that had been identified. We argue that the systemic response evidenced largely by the empty coffers and the absence of innovation-crisis stewardship indicates a concentration of executive authority in an overwhelmingly clinical, militarized, and potentially damaging supply zone.

**Methods:**

The analysis is grounded in the reviewed literature on health system governance and the system's lens that has been used to decenter public knowledge on the surface of recurring, persistent, and self-reinforcing institutional logics. The analysis is further informed by governance and decision theory, particularly the principal-agent theory, to identify likely facilitative mechanisms.

**Results:**

Looking at the medicines shortage with this lens, there was a steady, predictable, and alarming succession of steps: signals were allowed to build, no corrective measures were taken, and emergency procedures were instituted and outside assistance was used, in particular, to stabilize the situation. Some of the more immediate and focused assistance from the United Nations was additional evidence of the absence of medicines caused by the routine care of the government. Central Medical Stores and hospital-level accountability were being discussed, and the absence of integration and accountability among the many professional areas likely caused delays in critical situations. The HIV governance arrangements of Botswana at that time illustrate an improvement in the ability to both detect and respond to the situation in a more consolidated fashion.

**Conclusion:**

The governing stress test of the 2025 episode is more significant than a one-off operational failure. Aimed at preventing the same situation from happening, further measures are needed that integrate clinical approval with management and financial administration, reinforce emergency measures, and create an environment of learning to influence the medicines system. Although some of the planned initiatives may have an impact, the authority and accountability gaps will continue to be the main issues that concern the leadership.

## Highlights

- Botswana's medicines crisis in 2025 was a governance stress test and not an isolated supply-chain failure. It revealed how leadership structures, decision rights, and accountability arrangements endure sustained fiscal and operational pressure.
- Resilience in health systems demands more than just political visibility and emergency response mechanisms. It requires ongoing stewardship with explicit mandates and the ability to take action on early warning signs before situations escalate into crises.
- Some of the historical successes of the HIV scale up in Botswana were a result of committed political, administrative and technical coordination. This demonstrates that distributed leadership and disciplined coordination are more important to the performance of a system than individual leadership.
- Over-concentration of executive stewardship within a single professional worldview creates epistemic blind spots and weakens medicines governance by marginalising critical non-clinical expertise in procurement, financing, and supply-chain management.
- Redesigning stewardship for complex health systems requires multidisciplinary leadership, stronger decision intelligence, and institutionalised learning, particularly as countries integrate HIV and non-communicable disease (NCD) services under conditions of fiscal constraint.

## 1 Introduction: from exceptional performance to a 2025 stress test

Botswana has often been presented in global health-policy discussions as an example of what sustained political commitment and relatively stable public administration can achieve in an African setting (1, 2). As an upper-middle-income country with a small population and a strong tradition of state involvement in service delivery, the country built a reputation for relatively reliable public-sector health services. This reputation was further strengthened in the early 2000s during the rapid scale-up of antiretroviral therapy, when political coordination and formalized partnerships helped to enable large-scale implementation (3, 4). Over time, these developments fed into a broader perception of Botswana as a relatively stable and capable health-governance system.

The 2025 declared Public Health Emergency disrupted this stability (5, 6). Some patients with chronic illnesses were asked to return later for repeat prescriptions, and access to essential medicines was restricted in multiple districts. There were significant consequences beyond access to medicines. Front-line health workers interrupted elective procedures and raised concerns about the safety of patients. They also expressed concerns about their own safety as well as the safety of other staff members, and the burden that is being placed on staff as a result of the increased demands on the services (5–7).

There was no shortage of public explanations. The focus was on delayed payment to beaconing suppliers, problems with procurement and increased fiscal pressures as a result of decreased revenue from diamonds (8–10). These explanations were relevant and significant, but they were totally inadequate as an explanation for the failure to respond to the warning signs that were evident over a number of years.

The crisis served as a stress test for governance systems. The focus is on leadership systems, and more specifically, how poorly leadership systems are able to respond to the pressure of crisis situations. Questions arise regarding decision-making power, escalation pathways, and accountability during times of pressure on the institution.

Incorporating systems thinking, the analysis decouples the shorter-term events from the longer-term underlying trends that have been formed over time (11–14). The analysis draws attention to the institutional frameworks that shape the ways in which risks are recognized, communicated, and acted on. Therefore, the crisis is analyzed within the context of Botswana's changing fiscal and donor landscape (8–10) as well as within the context of the earlier leadership structures that facilitated the coordination of the rapid expansion of HIV treatment programs (3, 4).

What is being argued here is of a structural nature rather than a personal one. The focus is not on the dedication or the skills of the individuals, but rather how executive power and leadership stewardship are positioned within the health system. Financing medicines, oversight of procurement and balancing the supply chain is of a clinically informed nature. It requires the integration of control and management of the operations and contracts along the supply chain with clinical expertise (22, 23).

These pressures become more apparent during times of financial constraint. Leadership systems that concentrate power and authority in a narrow fashion may become exposed to the risk of being unresponsive to emerging risks in the system. In contrast, leadership systems that focus on distributed power and authority in a balanced fashion, may improve the system's ability to cope under pressure (24, 28–31, 42–44).

In health systems, Clinical leadership is crucial in upholding the standards of care, determining the priorities of services, and ensuring the safety of patients. However, challenges may arise when too much executive authority is concentrated within a single profession. In the absence of a clinical viewpoint, it's understood that leadership will focus on the continuity of services, and on the immediate clinical requirements of the patient. In the context of procurement, the oversight of the budget, and the management of supply chains and contracts, within the framework of core governance, these functions will become, at best, routine administrative functions (15–18).

Questions begin to emerge around decision-making authority, escalation processes and accountability during periods of institutional strain. Eventually, this will build institutional blind spots. The system doesn't expect clinical leaders to be any less competent or committed to the system. However, the intricacies of a health care system challenge the ability to bring together, and inter-relate, various components, including finance, logistics, administration, clinical delivery, and management.

A health system's robustness is able to secure the requisite balance across the various governance domains. Clinical governance is important, and more so, is the balance of clinical governance in the presence of the financial governance systems, operational governance systems, and accountability systems (17–20). The management of contracts, procurement of medicines, monitoring of stocks, and management of health system performance is not the routine practice of administration. The management of these functions will determine the timely and adequate delivery of medicines to a health care facility and the early identification of supply disruption.

Governance systems have an inherent risk that issues within them will be compounded, and can, at best, remain undetectable, until a critical failure is reached in the health delivery services. This study therefore applies a theory to conduct an analysis of the medicines emergency in Botswana in 2025. The analysis seeks to explore the impact of leadership on the ability of the health system to respond and adapt under pressure using systems thinking and a governance framework.

## 2 Materials and methods

The study employs a qualitative, documentary policy analysis approach, which is consistent with existing research in health systems and governance. The analysis is based on publicly available documents and media reports from the crisis and established health systems governance literature. A systems thinking approach enables the distinction of visible situations from the repetitive patterns and the deeper institutional frameworks that govern decisions. The analysis is supported by the theories of governance and decision making, including the principal-agent relations, and aims to illuminate likely mechanisms without asserting conclusive causal arguments.

This study follows a qualitative documentary policy analysis design, consistent with approaches used in health systems and governance research.

The analysis draws on publicly available documents, media reporting from the period of the crisis, and established health systems governance literature. A systems lens is used to separate visible events from recurring patterns and the underlying institutional structures that shape decision-making. Concepts from governance and decision-science (including principal–agent dynamics) guide the explanation, with the purpose of clarifying plausible mechanisms rather than claiming definitive causal proof.

By relying on systems thinking to differentiate moments from the patterns and the frameworks of the institutions that govern decisions and the escalation of risks, the episode is contextualized within Botswana's evolving fiscal and donor environment and in the context of preceding leadership formations that allowed for orchestrated implementation during the HIV expansion period.

To investigate this assertion, a theory-based policy analysis is applied to the 2025 medicines emergency. It employs systems thinking and governance frameworks to examine how the configuration of leadership influences the capacity for escalation, adaptation, and correction.

### 2.1 Study design and classification

This article considers itself a qualitative documentary policy analysis. It is not a narrative literature review. It was not meant to be an analysis of the existing scholarship. The focus was how the Botswana 2025 medicines crisis was portrayed in public documents, official reports, media reports and the relevant health systems governance literature. These documents served as evidence for the events, public responses, institutional reactions, and emerging governance controversies.

The focus of the analysis was a concrete policy episode: the 2025 public health emergency caused by the paucity of essential medicines and medical supplies in Botswana. It relied on documentary material to reconstruct the visible micro-sequence of events, spot repetitive governance related phenomena, and decipher the order of the responses of the authorities. The article thus provides the first structural health systems governance analysis using these materials and not simply a descriptive review of the medicines governance literature.

### 2.2 Document selection and analytical approach

The analysis was based on publicly available documentary materials produced from 2017 to early 2026. The selected timeframe aimed to analyze both the urgent 2025 medicines crisis and previous governance issues regarding procurement, service delivery, institutional accountability, and system coordination in Botswana's public health sector. Information was obtained from government sources, policy documents, media, government reports, assertions from professional associations, and peer-reviewed health and governance literature.

Carefully constructed searches of government websites, newspaper archives, institutional publications and academic databases yielded pertinent documents. Search terms consisted of several phrases: “Botswana medicines shortage,” “public health emergency Botswana,” “Central Medical Stores Botswana,” “medicine stock-outs Botswana,” “health governance Botswana,” “procurement crisis Botswana,” and “health system leadership Botswana.”

Documents that contained in-depth commentary on various topics such as governance, procurement, and supply chain issues, institutional accountability, leadership during the 2025 crisis, and emergency response strategies were also selected for analysis. Any materials that lacked a clear source or informational value, including those that focused exclusively on clinical issues, were excluded from the analysis.

The study did not equalize all documentary sources as evidence. Public statements and press reports were interpreted cautiously and assessed in conjunction with policy documents, institutional reports, and existing health systems research. If applicable, reported events were cross-verified with multiple sources to minimize the reliance on one account.

The interpretive framework for the analysis was derived from a transdisciplinary perspective. Documents were compared in order to capture and record the essence of governance, institutional discord, the failure to escalate, and leadership dynamics during the crisis.

The analysis elucidated the emergent properties of governance associated with the crisis and the subsequent responses. The interpretation invokes systems thinking and governance to describe the visible phenomena as well as the underlying recurrent patterns and structural conditions impacting the decisions and the institutional responses. The main aim of the analysis was to understand the impact of governance on the crisis and the responses it generated.

## 3 Results

### 3.1 The 2025 medicines emergency: events, patterns, and underlying structure

By 2025, almost all of Botswana's public health facilities had no essential medicines. Patients from multiple districts reported that they were told to return to the facility to collect their routine medicines. Disruptions to the continuity of treatment for chronic conditions occurred at all levels of the health system, including both primary care clinics and referral hospitals.

The most apparent symptoms of the crisis were caused by delays in purchasing, late supplier payments, and ongoing inventory shortages of key medicines. These issues garnered public and political interest and required emergency actions (3–6). To address these persistent issues, the Government of Botswana declared a public health emergency because the shortages of critical medicines and medical supplies disrupted routine health services and raised the concern of the Government of Botswana over the safety and health of citizens. Emergency measures included extraordinary procurement, additional funding, and an emergency procurement operation of military distribution to address the gaps in the availability of medicines (3).

The documentary record also shows that the 2025 crisis did not start from zero. Previous reports to Princess Marina Hospital had noted chronic problems such as high volumes in the system, delays, inadequate staffing, poor referral and health system accreditation, and drug management issues related to supply chain centralization (21–23). Commentators had also called for stronger controls, including an independent health ombudsman, to address persistent discrepancies of unreported failures and the absence of corrective actions (13).

The crisis was allowed to deepen to the point where the emergency response of United Nations agencies was activated. Media reports indicated that this support aimed to stabilize critical health systems in the face of high levels of system pressure (28). This emergency support was only a short-term solution and illustrated that routine external mechanisms could not mitigate the crisis before it reached an emergency level.

Central Medical Stores became the center of public discourse.In September 2025, news outlets reported that head leaders of CMS defended their team against claims of corruption and inefficiency, and reported that breakdowns were due to deficiencies in the Ministry of Health's oversight and direction (15, 29). These comments came after the President announced the formation of a task team to investigate CMS and ongoing shortages based on attributed public concerns (29).

The public comments that followed suggested that the breakdown of responsibility was ill-defined. The focus shifted from CMS to the Ministry of Health, suppliers, and the governmental systems as a whole. Instead of coming to a single, clear definition of the responsibility, the crisis laid bare the demands of multiple contending claims and the interdependencies of these claims. In situations where the scope of demands and claims is not defined, the purpose of the investigation is to create a public account of the issues, even if it does not achieve anything effectively.

There were numerous warning signs in the various documents before the state of emergency was declared. These included a lack of demand forecasting, partial implementation of stock-monitoring systems, delayed stock deliveries, and inconsistent enforcement of procurement and supply contracts (9, 16–19). Even stock controlling pharmacists and nurses warned of supply shortages and delivery delays.

When taken as a whole, the findings outlined the breakdown of the supply chain as more than the disruption of the supply chain as an emergency. There was significant, evident disruption of the supply chain as an emergency, but unresolved, cumulative demands theoretically shifted the work of the Ministry. Short-term demands in the Ministry eased pressure, but did not address the system's deficiencies of supply chain management, coordination, accountability, oversight and stakeholder engagement (30–33).

The way authority, expertise, and accountability were structured also exacerbated the crisis. Centralized procurement, disparate information exchanges between facilities and central agencies, and insufficient delegation of authority to supply-chain experts resulted in a loss of the ability to manage the demands of day-to-day functions when combined with the pressures of time (9, 10, 17, 20). Financial pressure caused these weaknesses to be more apparent, but the evidence available suggests that financial pressure alone could not have caused the crisis. The shortage of funds became acute when financial pressure, procurement weaknesses, poor information flows, and unassigned accountability were allowed to interact over an extended period.

### 3.2 Professional dominance and stewardship risk

Another of the challenges is how professional authority is structured within health governance systems. In numerous health systems, clinical leaders are the central and, in most instances, the most visible participants in the decision-making process. This is quite rational, given the range and importance of medical judgment in shaping the process and outcome of the delivery of health services and the care of patients. However, complex health systems are also heavily reliant on procurement, finance, and the administration of contracts as well as the management and administration of related logistics.

During the 2025 medicines crisis, public discussions focusing on shortages usually emphasized the continuity of clinical services and the emergency response. There was little focus, at least initially, on governance frameworks relating to procurement, payment systems, and the management of suppliers and other administrative coordination puzzles. As the crisis continued to evolve, it became increasingly challenging to disentangle the operational disruptions relating to the availability of medicines and the dimensions of governance.

The information analyzed in this case study does not demonstrate a causal relationship between a single professional group and the crisis. What appears to be the case is that a decision-making concentration of a single professional group resulted in a crisis, and a variety of professional groups may be preferred to justify budgetary procurement and supply management decisions.

When the supply chain is disrupted, delayed payment to suppliers, unstable contracts, and a backlog of procurement can lead to a lack of service delivery in fiscally constrained environments. Beyond clinical judgement, the balance of administrative and financial coordination plays a critical role in the continued delivery of care. Payment delays, procurement bottlenecks, and poor coordination across different parts of the health system are among the reasons cited for the 2025 shortages (24–27).

The focus here is on governance systems and not on individual actors. In complex health systems, performance is suboptimal when a variety of different types of professional governance and leadership are integrated. Pharmacists, procurement professionals, finance professionals, public service professionals, and clinicians each have a role to play in the health system when their professional functions are integrated.

When coordination in certain areas is limited, risks tend to build up behind the scenes and eventually create service disruptions. We might interpret Botswana's 2025 disruptions to service delivery as an inability to manage the complexities of governance. The disruptions revealed a lack of integration between management, procurement, and finance. The 2025 disruptions put excessive strain on governance and revealed, progressively, the limits of flexibility as resource shortages deepened.

### 3.3 Political economy and donor transition: why fiscal stress alters governance behaviour

Botswana's 2025 Medicines Crisis was primarily the result of fiscal constraints. The medicine shortages happened in a context of poor public finances, growing expenditure pressures, and diminishing economic conditions. The 2025 Medicines Crisis was composed of delays in payment, a backlog in procurement, and an increased uncertainty of supply. Under normal conditions, each of these factors would have caused relatively insignificant disruptions. However, fiscal constraints led to more significant disruptions, and this is also where Botswana's 2025 Medicines Crisis has its roots (34, 35).

For a Government, the failure to make payments on time is seen as either an administrative or a cash-management failure. The failure to pay suppliers can rapidly deteriorate the health system. Suppliers may decide to discontinue delivery, offer credit on more generous terms, or offer delivery to those clients more likely to pay. Facilities tend to have a stable supply of stock, and employees, on the frontline of health delivery, have to cope with a lack of stock and a poor supply of health-related materials. The crisis was the result of fiscal constraints; however, it should also be understood as the result of the procurement system, fiscal constraints, contract management, and coordination that was taken to a level that the system could no longer sustain(36, 37).

Delays in procurement combine with late payments to suppliers, poor stock visibility, and lack of clear escalation pathways, weakening the procurement system. A delay in one area causes stress in others. When enough of these weaknesses combine, the procurement system is no longer able to reestablish equilibrium, and the procurement system begins to affect patient care.

Botswana's donor transition is an additional challenge. During the HIV rapid scale-up, external donor partners helped strengthen financing, technical assistance, procurement system, and program coordination. As those arrangements changed, more of the responsibility was transferred to the domestic actors (38, 39). While this encouraged the domestic ownership, it increased the pressure on the local systems for planning, financing, procurement, oversight, and performance monitoring.

The potential danger is that, while transference may enable progress, it does not automatically supply capacity. Domestic ownership requires institutions that are prepared to assume functions that, until now, have partially been funded from outside. Where such functions have not been sufficiently resourced, the gap is likely to be most noticeable in the case of constrained fiscal space and increased demand for pharmaceutical products (40–42).

The crisis of 2025 needs to be analyzed from both perspectives. Issuing a new currency was only one part of the story. It was also not only the reconfiguration of the leadership structure. A more plausible answer lies in the interaction of procurement gaps, strained fiscal resources, weak supply lines, and the control of order. This approach provides a more comprehensive account of the crisis and avoids the temptation to reduce the answer to a simple description of a complex simultaneous failure of a system.

### 3.4 Historical evidence on leadership and success: what Botswana's HIV-era ecosystem teaches

The most important health policy achievements in Botswana were not the attributable accomplishments of individuals. They were the result of a carefully considered alignment of political power, administrative control, and technical skills to support large scale policy and program implementation (1, 2, 11, 12). Coalition building across government, disciplined control of delivery systems, and the strategic placement of external funding and resources sustained government reform during the first response to the HIV/AIDS epidemic. The correct lesson of governance is that the success of the first response was due not just to the political leadership of the response, but the presence of dynamic systems of distributed authority and governance routines at each level of the system that encouraged ongoing learning, coordination, and adaptive management (7–10).

#### 3.4.1 Building the National AIDS Co-ordinating agency (NACA): directive power, coalition governance, and managing professional boundaries

Establishing and entrenching the various national HIV/AIDS coordinating mechanisms and structures [including the National AIDS Co-ordinating Agency (NACA)] illustrates the ability of directive leadership to implement cross-sector accountability in difficult and complex programs (2, 11, 12, 22). These mechanisms and structures provided a practical means of focusing the disparate ministries, the donors, and the implementing agencies on the same goals and aspirations.

At the same time, Botswana shows how coordination structures may become fragile if some elements, such as mandates, decision rights, and incentives, are unclear. In those circumstances, lack of clarity over who has decision rights leads to contestation among professional groups, increasing the cost of coordination and resources needed for decision making, and creating inertia instead of speeding up the process (7, 8, 10, 25).

Coordination was most effective when political sponsorship created a sense of urgency and administrative leadership ensured process integrity. It was also effective when technical actors turned evidence into actionable routines (1, 2). This synergy was most effective, as it reduced transaction costs and minimized turf disputes. From the perspective of medicine, it is directly applicable to the procurement system and HIV-NCD (non-communicable diseases). Integration and the management of cross-professional collaboration will be needed, and the impact of unresolved boundary disputes will intensify under financial constraints (7–12).

This configuration has significance beyond HIV. It also represents a governance model that could be applied to many other areas of a system where multidisciplinary collaboration and integration are essential and where decision making is unambiguous. We do not mean to suggest that resources from the HIV era can be attained anew, but rather the governance components of clear roles, shared authority, and coordination, are useful in the current context of diminishing resources.

#### 3.4.2 The Joy Phumaphi era: boundary-spanning stewardship and disciplined execution

The period associated with the leadership of Joy Phumaphi is often cited for turning political commitment into the delivery of results (1, 2, 26). Analytically, its significance lies in bridging the gap between the rarely coordinated clinician and economist, the donor and civil servant, the central planner and the frontline worker. These links reduced bottlenecks and expedient learning when the early decisions were to the disadvantage of those on the ground (7, 8, 10, 25).

Gradual and steady credibility has emerged from the way priorities were clearly communicated and acted upon. Performance standards were evident, but delays caused by the lack of implementation were not viewed as personal failings. Rather, they were viewed as the need for system adjustments. The strategic focus of partnership management of external resources intended to strengthen national plans, not replace the country's governance or accountability (1, 2). Integrating these aspects during a time of rapid system growth developed appropriate feedback loops and aligned incentives, increasing system resilience. Integrating the partnership management, performance standards, and the consideration for the lack of implementation during rapid system growth also enhanced system resilience (7, 8, 10).

#### 3.4.3 Ruth maphorisa and NCD policy: adoption, adaptation, and institutional discipline

Ruth Maphorisa's contribution to Botswana's non-communicable diseases (NCD) policy is an example of a different but complementary form of leadership: maintaining a focus on organizational purpose in the context of increasing complexity. As Permanent Secretary for the Ministry of Health and Wellness, Maphorisa found ways to move NCDs beyond a clinical focus and treat them as a cross-cutting policy priority. This was done using the Botswana Multi-Sectoral Strategy on the Prevention and Control of Non-Communicable Diseases. In this context, the focus was on using international standards and WHO “Best Buys,” to allow Botswana's NCD institutions to work within and use their own systems (27).

The main obstacle was not the development of a policy; it was the sustainability of the policy. Sustaining cross-cutting compliance and financing became difficult as political priorities shifted and economic pressures increased. This reinforces a deeper insight into systems: the focus of policies needs to change to take a long-term view of prevention so that the harmful effects of short-term political and economic pressures do not undermine them. In this regard, the NCDs experience substantiates the claim of this paper that in health governance, administrative discipline, and the capability to make decisions and learn, are as important as having technical expertise and knowledge.

#### 3.4.4 From political to administrative stewardship: policy intelligence across leadership roles

Leadership in the health sector of Botswana demonstrates that complex and cross-cutting portfolios are beyond the scope of clinical authority and expertise. Political leadership, in the examples of Joy Phumaphi and Gloria Somolekae, demonstrates that clinical authority can also be instrumental in developing cross-cutting policy, rather than signalling intent. In their cases, leadership legitimized difficult reform decisions, and enabled the cooperation of actors who otherwise would have acted independently.

This kind of stewardship is not better or worse than technocratic governance. The real question is whether political power is backed by institutions that have the ability to learn, adapt and coordinate over the long term (10, 25).

Senior administration figures Ruth Maphorisa and Grace Muzila draw attention to the civil-service leadership role in the integration of budgets, contracts, information systems, and accountability. Strong administrative capabilities mean that systems will more likely use performance data for decisions, for viewing procurement and budgeting as decided strategic functions, and for recognizing and including non-clinical personnel as co-stewards (7, 8, 10, 25). This capability is crucial to meet Botswana's challenges in medicine and chronic care to reconcile the demands of service continuity and system learning for the long term while working within the limitations of available fiscal resources (9, 10, 16, 17).

### 3.5 Failure modes and the medicines crisis: when stewardship fragments

Fiscal tightening alone cannot explain Botswana's recent medicine shortages. Instead, these shortages point to a slow weakening of the leadership and coordination structures that previously helped the system learn, work together, and correct problems early. Unlike in the HIV era, when political, administrative, and technical domains deliberately distributed authority, the contemporary governance environment has become increasingly fragmented. As a result, early warning signals were detected but were not translated into decisive, system-wide action (28, 29).

At the core of this failure mode is the narrowing of decision-making authority around clinical leadership without a corresponding strengthening of administrative and managerial capability. While clinical expertise is indispensable, medicine availability depends primarily on functions that are outside the clinical domain: procurement planning, contract management, inventory control, budget execution, and supplier relations. When these functions are weakly coordinated or insufficiently empowered within executive decision-making, system performance becomes fragile even in the presence of technical competence (20, 21, 24).

#### 3.5.1 The absence of escalation triggers: early warning signals

Evidence suggests that early signs of stress, including delays in supplier payments, procurement bottlenecks, and inconsistent inventory levels, were present prior to observed shortages. These signs, however, did not prompt the sort of escalation or cross-level coordination seen in earlier reform periods. Unlike HIV response that involved collective, retrospective reviews with performance deviations leading to swift corrections, contemporary governance structures seem to be less capable of transforming information into action (7–10).

This failure illustrates a management issue rather than a data issue. Data was available. However, the ownership and responsibility to act was unclear. There was no clear authority that had the power and the incentive to initiate collective action to close the gaps. Problems were dealt with tactically rather than systematically. There was a backlog of risks that were eventually serviced (28, 29).

#### 3.5.2 Professional silos and the limits of clinical dominance

The second reason for the crisis is the unresolved tensions of professional boundaries. As the clinical leaders took greater control of management, the non-clinical expertise of the supply chain, health, and public financing was both pushed to the side and brought in later to the decision-making processes. This inhibited the ability of the system to come up with solutions to the delayed payment and the resulting loss of trust from the suppliers. In the 2025 crisis, it was reported that delayed payments to suppliers and the backlog of contracts were seen as administrative issues to be resolved and were not of concern to management. By the time management acted on the issues, the disruptions were already being experienced (20, 21, 24).

It is not an argument against the concept of clinician management. However, if the issues are defined too narrowly, the lack of public health expertise will create structural problems, and the administrative issues will be neglected until they eventually cause a crisis.

The shortage of medicine indicates the kind of governance that discourages collective responsibility, cross-boundary problem-solving, and the ability to learn from experience (28, 29).

#### 3.5.3 From adaptive coordination to reactive management

Although some of the emergency actions of the medicine supply crisis may have helped resolve short-term demand, in the longer term, rebuilding resilience and restoring trust requires more than the absence of emergency actions. This is in stark contrast to the HIV response, where governance structures were designed to manage and absorb the governance challenges of the crisis. Over time, the governance of the HIV response was coordinated and shared leadership. The governance shifted to the management of governance challenges, and the response was more reactive, with management of crises rather than addressing the governance challenges through proactive routines and institutionalized practices (28, 29).

#### 3.5.4 The stewardship gap

The medicines crisis demonstrated not only the absence of resources, but also the absence of stewardship. The combination of authority and the expertise of governance demonstrated that, once the gap in resources was created, the weaknesses of the governance structures became apparent. Once administrative leadership begins to shift, and the non-clinical workforce is not employed as co-stewards, and the learning systems become ineffective, the strongest systems of governance begin to fray and the continuity of services is lost (7–10, 28, 29).

The lessons of the medicines crisis is that Botswana needs more stewardship, governance, and financing, and advanced forecasting tools, rather than more resources, governance, and financing. Leadership arrangements of clinical governance will integrate administrative discipline and a stewardship approach if the support functions are aligned. Without a stewardship alignment, warning systems are ineffective, and the governance and coordination system collapses, causing repeated avoidable crises.

## 4 Professional dominance and stewardship configuration in complex health systems

When clinical services are embedded in care, the management of services sustains governance. The issue is not regarding the importance of medical knowledge. The issue is the way authority is structured across a complex health system. If decisions at the highest level of management are dominated by one professional perspective, gaps will develop in areas that require other competencies. Oversight of purchasing, budgeting, logistics, and contracts are just a few examples (22, 23).

Each health system integrates multiple fields of expertise. Clinical care is just one system. So too is the management of purchasing and budgeting, of the flow of goods and information (sleep) (42, 43). No health profession is able to manage all of the components of this system. There usually are other management, financial, or operational systems that, although they are outside of direct clinical care, are highly stressed.

This argument shows how authority is shared and how non-clinical knowledge is brought into governance. Similar challenges may occur in any health system where executive authority is concentrated in one profession and other domains are broadly represented rather than fully staffed.

Institutional challenges are slow and build gradually through a lack of clear mandates, uneven authority, ineffective enforcement, and fragmented accountability. In highly vested professional systems, non-clinical staff struggle to influence decisions that create risks within their area of professional expertise (22). Coordinators in pharmacy roles may observe a destabilizing supply of medicines. Procurement staff may see the compromised contracts in the system. Finance staff may experience blocked payments. Concerns may not be escalated in the absence of clear pathways due to a lack of administrative professionalism, which is often perceived as secondary to the primary focus of the stewardship functions (37, 38).

Health systems gradually learn to live with instability. Risks are progressively accepted as the business as usual. When abnormal measures become the norm, governance is finally invoked to respond to disruptions that are obvious and hard to ignore (39). Regulative measures are replaced with crisis management. The same findings are reflected in the literature on distributed and collective leadership. When authority is distributed and not concentrated on one profession, health systems tend to be more effective (24). Good governance still promotes clinical leadership.

Rather, it supports it by keeping oversight of procurement, financial controls, and contract management, as well as accountability mechanisms, even during non-crisis periods (22, 23). Where attention and oversight arrangements become inconsistent, coordination may deteriorate, responsibilities may be contested, and the pace of an institution's response may slow, particularly during times of fiscal stress.

The same sort of problems can also be found in the research on the governance of medicines. A stock out is rarely the result of a single bad procurement choice. It is usually the result of a number of pressures building up over time. Poor forecasting, late payment, and poor information are coupled with inconsistent enforcement and fragmented accountability. These pressures can become cumulative (19, 23, 30, 32, 33). During the 2025 medicines crisis Botswana suffered from many of these governance problems.

Another challenge is how Authority and Ownership are organized in the health system. In some cases, the executive leadership is primarily associated with one dominant professional group, and the other technical professionals are mostly being drawn to the health system's leadership in the implementation of work. If this continues, leadership and decision making in the areas of expertise of procurement, health economics, public administration, and pharmacy are diminished, and these professionals are omitted from making critical decisions (24, 31, 34). The challenge therefore lies in the structure, rather than in the individual. While it is possible that Professional Concentration is stable, it is also likely that it can be more unstable when various pressures (fiscal, operational, and resource management) are experienced in the system. This would certainly describe the situation in Botswana in the health system governance crisis of 2025 (10, 14, 28, 29, 35).

### 4.1 Comparative lenses: what global system failures reveal about monoculture and learning

Governance crises of this nature are not unique to Botswana and the same phenomena are seen in many other high-income and low-income health systems. In a lot of these systems, it is possible to identify some of the warning signs, but they go unaddressed. Defensive culture sets in as accountability declines. Disruptive incidents that are made publicly known often trigger overreactions, when compared with how aggressively systems dealt with concerns during the early manageable stages (31, 36, 37).

Absence of information as the problem is the exception and not the rule. More often than not, the problem is with the flow of information across the institution, and whether the leadership willing and able to respond to early, uncomfortable, and inconvenient signals. If feedback is systematically deferred and treated as a challenge to the authority, the system will inevitably slow down (28, 29, 31).

The challenges that face the LMICs medicine system is not too dissimilar. Supply shortages emerge through several inter-related risks, and not just as a result of a single mistake.

Research from LMIC medicines systems points to similar pressures. Supply shortages usually emerge through several interacting weaknesses rather than one isolated mistake alone. Forecasting issues, procurement delays, inadequate contract enforcement, fragmented information systems, and deficient coordination tend to reinforce each other and gradually become compounded over time (19, 23, 32, 33). This is important for Botswana because it shows that the medicines crisis of 2025 should not be labeled solely as a result of procurement failure. It is more concerning the manner in which systems of governance perceive and respond to the pressure of the system rather than allowing disruptions to the system to reach critical points.

## 5 Was 2025 a leadership crisis? political stewardship, administrative capability, and accountability

Whether or not the phenomena of 2025 can be said to represent a crisis of leadership will always be a function of the definition of leadership you choose to adopt. If the definition of leadership is synonymous with the ability to respond to emergencies with the aid of visible leadership, then Botswana, without the aid of a leadership crisis, was able to respond to the failure of leadership through the visible and rapid implementation of extraordinary measures (4, 5, 10, 14). If you adopt a systems view, leadership is defined as the ability to respond to emergencies and the ability to perceive and respond to potential disruptions of the system. The ability to do all of this while the system is operational and to foster the ability to learn in the system is also the definition of leadership. If this crisis is looked at from a systems view, it was not simply an emergency that disrupted the system, but also a crisis that revealed the failure of leadership and the failure to account for the system.

There is a quiet yet significant impact that political stewardship has on system resilience. The way governing institutions are structured, the prioritization of fiscal policy, and the systems of accountability shape the system's capacity to absorb and manage shocks (4, 5, 10, 14). Supplier arrears and disruption of the procurement cycle are governance failures. They demonstrate a lack of budgeting discipline, a failure of cash-flow management, and the failure of the state to meet its contractual obligations to service providers. Decisions regarding executive appointments and the privileging of given types of expertise shape the governance framework and determine the way in which risks are understood and dealt with (31, 34).

The 2025 crisis, characterized in large part by. procurement, macrofiscal constraints, and governance design issues, was inevitable (4, 5, 10, 14, 19, 23). The purpose of this paper is to combine these strands together with a particular focus on the issues of governance design, mainly the structure of power and the control of expertise, to determine how much influence these factors had on the convergence of the various elements of the crisis and the lack of corrective action when the signs were clear (28–31).

## 6 Recommendations for strengthening stewardship and health-system coordination

Botswana's medicines crisis of 2025 is a perfect example of the failure to address an emerging problem that, instead, led the government to respond to an issue when it became a full-blown crisis of emergency. The case highlights the urgent need to design leadership adaptive to increasing pressures, the need for stronger leadership to tackle the challenges of dealing with the demands of governance, especially for managing pressures, uncertainty, and the need to coordinate across the different sectors of the health system. The problem was not the elimination of clinical leadership. The design of leadership and management in clinical systems, extensive clinical expertise, and management systems integrate to facilitate the development and management of the system before crises occur

First, governance of medicines needs to move towards slightly more balanced, multidisciplinary structures. Non-clinical leaders need to be heavily involved in regular decision-making. This includes procurement, finance, supply chains, and information system staff. Each part of the system has a type of expertise, and when thinking about supply chains, these roles are best seen as complementary and not secondary. From a clinical perspective, taking more responsibility for forecasting and managing suppliers, payments, and stocks, would help improve accountability before a critical shortage is reached.

Second, professional development of clinicians and other staff needs to be vertical and cross silo. The development of clinicians to become executives needs to encompass public administration, budget and finance, systems, and organisation. Conversely, clinicians and practitioners need to be actively involved in the development of the upper echelons of the profession, as opposed to being the ‘doers’ at the lower levels.

Finally, the crisis showed there needs to be a stronger organisational learning capacity. The information and data regarding the supply and systems from the facilities and pharmacies needs to be more integrated into formal structures and processes of governance in a timely manner.

Reliable reporting systems, visibility of stock conditions, and early governance processes are needed to respond to issues before they become widespread disruptions. Additionally, systems need to have environments in which operational issues can be raised without worrying that the news is going to be ignored or postponed.

Botswana's governance of HIV also provides lessons in the coordination of the management of chronic health conditions. HIV governance systems can provide the foundation for the management of other conditions, including the management of chronic non-communicable diseases (NCDs). Ultimately, coordination, funding and stronger regulatory and administrative mechanisms will be needed for the integration of these services. Expanding services without the requisite funding may simply move the burden from one part of the system to another without improving system performance.

## 7 Limitations

This research is based on evidence from a publicly available document analysis. This has the potential to obscure some of the internal discussions, informal decisions and/or confidential processes that may have contributed to the 2025 medicines crisis.

Because the publicly available documents do not encompass the full internal workings of the system, the research draws on other available data, including media reporting. The reporting was invaluable, as it documented the public discourse at the time of the crisis. It reported on the responses to the crisis, public explanations, timelines, and concerns raised by various stakeholders. That said, that the media is often incomplete and only partially reflects the available information at the time. For that reason, they have not been treated as stand-alone evidence. They were reviewed in conjunction with formal papers, publicly available communications, and the relevant health systems literature.

This analysis does not claim to pinpoint the single true cause of the crisis; rather, it attempts to pinpoint the role of synergistic relations among fiscal pressures, delays, and issues in the supply chain and stakeholder engagement, governance, and the failure to escalate appropriately. As such, the results presented here illuminate the public policy conundrum and should not be seen as an answer to the conundrum of all the necessary components.

## 8 Conclusion

Botswana's 2025 medicine emergency should not be considered an isolated system failure nor a retreat from public health commitment. Instead, it has shown the reality of the crisis governance framework, the vulnerability of leadership in a complex health system with simultaneous fiscal and disease pressures.

The country's previous successes in combatting HIV/AIDS have shown the possibility of effective health systems governance when the political, governance, and health system burdens are aligned, such as in the previous health governance interventions noted. We have the knowledge, the will, and the energy to govern; what we lack is the ability to configure our systems in such a way that we can manage the complexity, uncertainty, and ever shifting interdependencies. We seem to manage our systems for the unstable condition with the knowledge that we will not have system disruptions.

There is therefore a choice of options for Botswana. One option normalises the handling of crises as they occur, which will be silencing for the institution and the public the crisis fuels. The other option uses the 2025 crisis as an opportunity to adjust institutions, and strengthen multidisciplinary stewardship, decision intelligence, and the defense of long-term learning under constraints.

Taking this option would do more than support HIV-NCD integration or the availability of medicines. It would reinforce Botswana's position as a health system capable of delivering at a large scale, but also of reflecting, adapting, and leading in an increasingly complex environment of global health.

## Data Availability

Publicly available datasets were analyzed in this study. This data can be found here: The datasets analysed in this study are publicly available and can be accessed through the original sources cited in the reference list. These include government reports, publications from international organisations (such as the World Health Organization, International Monetary Fund, and African Development Bank), and publicly accessible media reports.All sources are fully referenced in the manuscript and can be accessed via the URLs provided in the reference list. No central repository or accession numbers apply to this study.
